# Prevalence of depressive symptoms and its correlations with positive psychological variables among Chinese medical students: an exploratory cross-sectional study

**DOI:** 10.1186/s12888-016-0710-3

**Published:** 2016-01-11

**Authors:** Meng Shi, Li Liu, Zi Yue Wang, Lie Wang

**Affiliations:** Department of English, School of Fundamental Sciences, China Medical University, 77 Puhe Road, Shenyang North New Area, Shenyang, Liaoning PR China; Department of Social Medicine, School of Public Health, China Medical University, 77 Puhe Road, Shenyang North New Area, Shenyang, Liaoning PR China

## Abstract

**Background:**

Knowledge about the prevalence of depressive symptoms among Chinese medical students and its related factors is rather limited. Understanding the correlates of depressive symptoms and the roles that positive psychological variables play in depressive symptoms is of vital importance for future interventions. The main objectives of this study were to investigate the prevalence of depressive symptoms and the integrated effects of resilience, hope and optimism on depressive symptoms among Chinese medical students.

**Methods:**

This multi-center cross-sectional study was conducted in June 2014. The questionnaires that consisted of the Center for Epidemiologic Studies Depression Scale (CES-D), Wagnild and Young Resilience Scale-14 (RS-14), Adult Dispositional Hope Scale (ADHS), Life Orientation Test-Revised (LOT-R), and socio-demographic characteristics, were distributed to students at four medical colleges or universities in Liaoning province, China. A total of 2925 medical students became the final subjects. Hierarchical linear regression analyses were used to explore the integrated effects of resilience, hope and optimism on depressive symptoms.

**Results:**

The prevalence of depressive symptoms among Chinese medical students was 66.8 % (CES-D ≥ 16). Resilience, hope and optimism were all negatively correlated with depressive symptoms and they accounted for 26.1 % of the variance in depressive symptoms.

**Conclusions:**

The high prevalence of depressive symptoms among Chinese medical students calls for special attention from all stakeholders, especially university authorities. Intervention strategies that focus on enhancing the positive psychological variables of resilience, hope and optimism can be integrated into depression prevention and treatment programs.

## Background

Depression is a major global public health concern, which has become the second highest burden in the age group of 15–44 years and is projected to become the second leading cause of early death and disability by 2020 [1]. It is also associated with elevated risks for several serious medical conditions, such as diabetes [2] and cardiovascular disease [3]. The depression in young adults is of great concern, not only because of the substantial burden of disease, but also due to the fact that adolescence and early adulthood is linked to the onset for a significant proportion of depression, and the persistence of the disease across life span [4, 5]. In addition, it has been well documented that depression has brought about many negative consequences to young people, such as poor academic performance, increased rates of substance use, and even suicide [6–9].

Medical school is a time of significant psychological distress for many students [10]. Previous studies suggest higher prevalence of depression among medical students in comparison to the general population [10–13]. It is revealed that the high prevalence of depression in physicians can partly be traced back to the medical education they receive [14]. Thus, the psychological well-being of medical students is of vital significance not only for the medical students themselves, but also for the quality of health care they will provide [15] and doctor-patient relationship in the future.

The high prevalence of psychological and mental health problems in medical students can be attributed to many factors, such as high stress in medical education [16] and lack of psychological resilience among the students [17]. Positive psychology is being increasingly used in the prevention and treatment of depression. Seligman et al. highlight that strategies of positive psychology generate positive coping resources, which in turn can alleviate depression and reduce their relapses [18]. Positive emotions, fundamental aspect of positive psychology, play a central role in the treatment and protection against the development of depression [17]. Resilience, hope and optimism are among the most studied positive emotions in the field.

Resilience refers to the capacity and dynamic process of adaptively overcoming stress and adversity while maintaining normal psychological and physical functioning [19]. With regard to the protective effect of resilience on mental health, prior studies mainly focused on the relationship between resilience and negative mental health, such as depression [20, 21]. The growing understanding of the neurobiology of resilience has significant implications for the prevention and treatment of depression [22]. Dispositional optimism is conceptualized as a relatively stable, continuous, and bipolar individual difference variable, reflecting a person’s overall expectations about future life events across different domains [23]. Dispositional optimism has been found to be negatively correlated with depression in cross-sectional studies [24] and function as a predictor of depression in longitudinal studies [25]. According to Snyder et al. trait hope is an enduring cognitive–motivational and goal-oriented construct, consisting of two distinct yet related elements that influence one’s perception that his or her goals can be achieved [26]. As a protective characteristic, hope facilitates the ability to overcome stressful life situations through engaged coping behavior and adaptive problem solving among university students [26]. It is also associated with decreased level of depression [27] and can prevent the development of depression [28, 29].

Although the prevalence of depression among medical students is high over the course of medical education, many students who are exposed to the same level of stress are less vulnerable to the disorder. Based on transactional stress theory [30], personality characteristics, such as resilience, optimism and hope, can influence appraisal process of individuals, which in turn mediates stressor-stress response. In addition, according to integrated resource model, individual psychological capacities coexist and are utilized as a collective rather than in isolation [31]. The three positive psychological constructs of resilience, hope and optimism are state-like and can be developed in both theory and practice. Prior studies have predominantly examined the effects of one of the three variables on depression in different groups. However, as-of-yet, the integrated effects of the three constructs on depression in medical students have not been explored. Given the huge population base in China, and the significant roles medical students are supposed to play in health care sector in the future, we conducted the current study with the following aims: 1) to investigate the prevalence of depressive symptoms among medical students in China; 2) to identify the demographic risk factors for depressive symptoms among Chinese medical students; 3) to examine the integrated effects of resilience, hope and optimism on depressive symptoms among Chinese medical students.

## Methods

### Study design and sample

A cross-sectional study was carried out in Liaoning province with a population of around 44,000,000, in northeast region of China in June 2014. All the four medical tertiary institutions in the province were included in the investigation, which were Shenyang Medical College, Liaoning Medical College, Dalian Medical University and China Medical University respectively. The medical education in China mainly consists of 5-year programs, 7-year programs and postgraduate education. As more medical students enroll in 5-year programs, 4 whole classes of clinical students in 5-year programs and 3 whole classes of clinical students in 7-year programs were randomly chosen from each institution based on academic year. The number of students in each class in Chinese medical schools usually ranges from 25 to 40 in both programs. Self-administered questionnaires were either distributed to the students in class or sent to the students of higher grades who were on the stage of clinical practice in teaching hospitals. A total number of 3500 questionnaires were distributed and 3095 (88.43 %) students returned the questionnaires in class or before the deadline. 170 invalid questionnaires were excluded and a pool of 2925 students (effective response rate: 83.57 %) became the final subjects.

The present study was approved by the Committee on Human Experimentation of Shenyang Medical College, the Committee on Human Experimentation of Liaoning Medical College, the Committee on Human Experimentation of Dalian Medical University and the Committee on Human Experimentation of China Medical University. Participation in the study was voluntary and written informed consent was obtained from the students or the minor students’ parents according to the Declaration of Helsinki (59^th^ WMA General Assembly, 2008).

### Demographic characteristics

Demographic information regarding age, gender, place of residence, study programs, academic year, and educational levels of both parents were obtained in this study. Place of residence was dichotomized into rural and urban areas. Study programs were divided into 5-year programs and 7-year programs. Educational levels of parents were categorized into primary school, middle school, university, and postgraduate school.

### Measurement of depression

Depression was measured with the Center for Epidemiologic Studies Depression Scale (CES-D) developed by Radloff [32]. It consists of 20 items (e.g., “I thought my life had been a failure”), and each item is scored on a 4-point Likert scale based on the frequency of depressive symptoms in the previous week, ranging from 0 (none or rarely of the time) to 3 (most or all of the time). The score from each item is summed to obtain an overall score, with higher score indicating higher level of depressive symptoms. Subjects scoring 16 and above were defined as depressed [30]. The CES-D demonstrated adequate reliability in Chinese university students [33, 34]. The Cronbach’s alpha for the scale in the present study was 0.89.

### Measurement of resilience

We assessed resilience with the 14-item Wagnild and Young Resilience Scale-14 (RS-14), which is one of the most reliable tools to measure resilience [35]. It is a 7-point Likert scale used in various age groups and different conditions [36–38]. Each item (e.g., “when I’m in a difficult situation I can usually find my way out of it”) is graded from 1 (strongly disagree) to 7 (strongly agree). Graded items are summed to generate a total score and lower scores indicate less resilience. The RS-14 has been validated among Chinese college students [39] and the Cronbach’s alpha for the scale was 0.94 in this study.

### Measurement of hope

Adult Dispositional Hope Scale, developed by Synder et al. [26], was used to measure hope level in the students. The scale contains 8 items (e.g., “I can think of many ways to get the things in life that are most important to me”) and 4 fillers. Each item is answered using an 8-point Likert-type scale, ranging from 1 (definitely false) to 8 (definitely true). The scores of the eight items are calculated to generate a total score and higher scores indicate higher level of hope. The scale has been used among Chinese college students with adequate reliability and validity [40]. In the current study, the Cronbach’s alpha value for this scale was 0.92.

### Measurement of optimism

Dispositional optimism was assessed with Life Orientation Test-Revised (LOT-R), developed by Scheier, Carver and Bridges [41]. LOT-R consists of 6 items (e.g., “in uncertain times, I usually expect the best”) plus 4 filler items. Each item is scored on a 5-point Likert scale, ranging from 1 (strongly disagree) to 5 (strongly agree). Higher scale score reflects a greater tendency to expect more positive outcomes. The scale has been used in Chinese college students [42, 43]. In the present study, the Cronbach’s alpha value for the scale was 0.67.

### Statistical analysis

All analyses were performed using SPSS statistical software for Windows version 17.0 (SPSS, Inc., Chicago, IL). All statistical tests were two-sided and the significance level was set at *p* < 0.05. Descriptive statistics of the demographic and psychological variables were indicated with mean, standard deviation (SD), number (N) and percentage (%) as appropriate. *T*-test and One-way ANOVA were used to compare differences in depressive symptoms among categorical groups. Pearson’s correlation was used to examine correlations among psychological variables. Hierarchical regression analysis was used to explore the effects of resilience, hope and optimism on depressive symptoms, as well as the interaction effects among the predictors. Standardized estimate (β), F, R^2^ and R^2^-changes (△R^2^) for each step were provided. Tolerance and variance inflation factor were used to check for multicollinearity.

## Results

### Characteristics of subjects

The basic characteristics of the medical students and the distributions of depressive symptoms in categorical variables are shown in Table 1. Among the 2925 students, 1028 (35.15 %) were males, while 1897 (64.85 %) were females. Their age ranged from 15 to 28 (M = 21.65, SD = 1.95). The prevalence of depressive symptoms was 66.8 % among the medical students. Compared with younger age group, the older students had a significantly higher prevalence of depressive symptoms (*p* < 0.05). Male students showed a higher prevalence of depressive symptoms than that of female ones (*p* < 0.05). The students studying in 5-year programs showed a higher prevalence relative to the students in 7-year programs (*p* < 0.05). Meanwhile, place of residence, paternal and maternal education did not appear to influence the prevalence of depressive symptoms (*p* > 0.05).

**Table 1:** Demographic characteristics and differences in depressive symptoms (*N* = 2925)

Variables	Number	Percent	CES-D (Mean ± SD)	*P*-value
Gender				
Male	1028	35.15 %	21.61 ± 9.34	< 0.001
Female	1897	64.85 %	19.68 ± 9.22	
Age				
15–21	1406	48.07 %	19.48 ± 9.58	< 0.001
22–28	1519	51.93 %	21.19 ± 8.97	
Place of residence				
Urban area	1809	61.85 %	20.33 ± 9.50	0.820
Rural area	1116	38.15 %	20.41 ± 9.00	
Paternal education				
Primary school	301	10.29 %	21.12 ± 9.40	0.210
Middle school	1450	49.58 %	20.05 ± 9.05	
University	1044	35.69 %	20.48 ± 9.67	
Postgraduate school	130	4.44 %	21.08 ± 8.96	
Maternal education				
Primary school	469	16.04 %	21.03 ± 9.08	0.365
Middle school	1524	52.10 %	20.16 ± 9.13	
University	841	28.75 %	20.33 ± 9.68	
Postgraduate school	91	3.11 %	20.56 ± 9.92	
Study programs				
Five years	1738	59.42 %	21.29 ± 9.18	< 0.001
Seven years	1187	40.58 %	19.00 ± 9.33	

CES-D: the Center for Epidemiologic Studies Depression Scale

### Correlations among the psychological variables

The means, standard deviations and the results of Pearson correlation analyses are presented in Table 2. Results revealed that all variables were significantly correlated with each other and were in the expected direction. Specifically, depressive symptoms was negatively correlated with all the positive psychological variables (resilience: *r* = −.0468, *p* < 0.01; hope: *r* = −0. 451, *p* < 0.01; optimism: *r* = −0.401, *p* < 0.01). In addition, resilience was highly related to hope (*r* = 0.721, *p* < 0.01) and moderately related to optimism (*r* = 0.392, *p* < 0.01); while hope was moderately associated with optimism (*r* = 0.370, *p* < 0.01).

**Table 2:** Means, standard deviation (SD) and correlations of psychological variables

Variables	Mean	SD	1	2	3	4
1. Depression	20.36	9.31	1			
2. Resilience	69.33	15.66	−0.468^**^	1		
3. Hope	42.43	10.41	−0.451^**^	0.721^**^	1	
4. Optimism	22.35	3.42	−0.401^**^	0.392^**^	0.370^**^	1

Depression: the Center for Epidemiologic Studies Depression Scale (CES-D); Resilience: Resilience Scale-14 (RS-14); Hope: Adult Dispositional Hope Scale (ADHS); Optimism: Life Orientation Test-Revised (LOT-R). **p < 0.01 (two-tailed)

### Hierarchical regression analyses

The results of the hierarchical regression analyses to explore the integrated effects of resilience, hope, optimism on depression and the interaction effects among the predictors are shown in Table 3. Because of the significant effects of age, gender, and study programs on depressive symptoms, these variables were entered into step 1 as control variables when depressive symptoms was dependent variable. As demonstrated in the table, resilience, hope and optimism as a whole accounted for 26.1 % of the variance in depressive symptoms in step 2. Resilience (β = −0.223, *p* < 0.001), hope (β = −0.195, *p* < 0.001) and optimism (β = −0.227, *p* < 0.001) were all negatively related to depressive symptoms.

**Table 3:** Results for hierarchical regression analyses of variables predicting depressive symptoms

Variables	Step 1 (β)	Step 2 (β)	Step 3 (β)		
			Model 1	Model 2	Model 3
Step1					
Age	0.116**	0.033*	0.035*	0.033*	0.035*
Gender	−0.101**	−0.047**	−0.049**	−0.050**	−0.051**
Study programs	−0.141**	−0.064**	−0.068**	−0.065**	−0.065**
Step2					
Resilience		−0.223**	−0.233**	−0.234**	−0.221**
Hope		−0.195**	−0.199**	−0.189**	−0.200**
Optimism		−0.227**	−0.222**	−0.229**	−0.228**
Step3					
Resilience × Hope			−0.054**		
Resilience × Optimism				−0.070**	
Hope × Optimism					−0.070**
F	38.884**	207.706**	180.266	182.077	182.151
R^2^	0.038	0.299	0.302	0.304	0.304
△R^2^	0.038	0.261	0.003	0.005	0.005

Resilience: Resilience Scale-14 (RS-14); Hope: Adult Dispositional Hope Scale (ADHS); Optimism: Life Orientation Test-Revised (LOT-R). *p < 0.05 (two-tailed); **p < 0.01 (two-tailed)

The interaction effects among the positive psychological constructs were explored in step 3. As shown in the table, the interaction between resilience and hope in model 1 (β = −0.054, *p* = 0.001), the interaction between resilience and optimism in model 2 (β = −0.070, *p* < 0.001) and the interaction between hope and optimism in model 3 (β = −0.070, *p* < 0.001) were all significantly associated with depressive symptoms, which accounted for an additional 0.3 %, 0.5 %, 0.5 % of the variance in the symptoms above and beyond what was explained by the covariates and main effects. Tolerance (range: 0.940–0.997) and variance inflation (range: 1.003–1.064) did not indicate a multicollinearity problem.

## Discussion

The present study investigated the prevalence of depressive symptoms and the integrated effects of resilience, hope and optimism on depressive symptoms among Chinese medical students. It is one of the largest multi-center epidemiologic studies on depressive symptoms in medical students. The results showed that the prevalence of depressive symptoms among Chinese medical students was as high as 66.8 %, which was considerably higher than 44.2 % among Chinese university students [44]. Previous studies suggest that the prevalence of depressive symptoms or depression in medical students is high [11–13]. Yoolwon Jeong et al. reported that 37.1 % of medical students in South Korea suffered from depressive symptoms [11], and Givens and Tjia revealed that the prevalence of depression among American medical students was 24 % [12]. In a systematic review by Hope and Henderson, the prevalence of depression among medical students in English-speaking countries outside North America ranged from 6.0 to 66.5 % [13]. Meanwhile, it is worth noting that the prevalence among Chinese medical students was quite similar to that in Chinese doctors (65.3 %) [45]. The high prevalence of depressive symptoms among Chinese medical students can be attributed to the tough environments inherent in medical education [16]. Medical students need to learn to face death in a professional context. However, many of them are not well prepared for end-of-life care and dealing with patient death [46], and some students develop death anxiety, which has been found to be associated with depression [47]. In addition, depressive symptoms have been shown to be strongly correlated with sleep deprivation, which is common in medical trainees [48]. Furthermore, in recent years, China has witnessed increasing reported numbers of serious doctor-patient conflicts, which are likely to exert negative influences on concerns about their future among the medical students.

Older medical students were found to be more susceptible to depression compared with younger ones, which is consistent with the finding in Chinese University students [49]. This may be explained by the fact that older students have to face more stressful events, such as clinical practice, employment, financial burden, and marriage pressures. Contrary to our result, some other research demonstrates that younger medical students are more vulnerable to depression [50].

The higher prevalence of depressive symptoms in male students in the study deserves attention. The two systematic reviews of depression among medical students in U.S. and Canada as well as in other English-speaking nations have yielded mixed result. While some studies demonstrate that female medical students are more susceptible to depression, others show no significant gender difference or higher prevalence in male students [10, 13]. The social-cultural factor may partly explain the result in the present study. Deeply rooted in Confucius thought, males in China still play a dominant role in both family and social life. Although women have gained equal legal rights in society, in Chinese culture today, men are usually considered as the pillar of family in many aspects. Thus, they are often confronted with more stressors than their female counterparts.

The students in 5-year programs had a higher prevalence of depressive symptoms than those in 7-year programs. The following two reasons may account for the difference. Firstly, better teaching and research resources are more accessible to the students in 7-year programs, compared with those in 5-year programs. This may result in inferior feelings for the latter students from time to time. More importantly, the prospect of landing satisfactory positions upon graduation for students in 5-year programs is much dimmer, as most high-quality hospitals in big cities in China today only recruit candidates with master degrees or above. Therefore, a relatively high proportion of 5-year programs students have to prepare for postgraduate school entrance examination to further their study, which can bring about huge stress.

Another important finding of the current study was that the three positive psychological variables of resilience, hope and optimism were all negatively correlated with depressive symptoms among Chinese medical students. The three variables accounted for 26.1 % of the variance in depressive symptoms. Prior research has revealed that each variable of resilience, hope and optimism is either negatively correlated with depression or functions as its predictor [20–22, 25, 27]. In the current study, these three constructs were shown to be independently related to depressive symptoms when they were entered into regression together, indicating that resilience, hope and optimism as a whole might better alleviate depression in medical students. In addition, three groups of interactions among the three positive psychological constructs were revealed in the study. Specifically, hope was shown to have a significant and negative moderating role on the relation between resilience and depressive symptoms, such that as level of hope increased, the association between resilience and depressive symptoms decreased in the medical students. Prior research has shown that hope, as a protective characteristic, functions as a moderator of the association between negative life events and depressive symptoms in an ethically diverse sample of college students [51]. Additionally, optimism was shown to have the same level of negative moderating effects on the independent associations of resilience and hope with depressive symptoms, such that as level of optimism increased, the association of resilience and hope in relation to depressive symptoms decreased in the medical students.

As positive psychology has been proved to have significant effects on the prevention and treatment of depressive symptoms [18], the results of the present study deserve attention in terms of depression prevention and reduction in medical students. Theoretically, a higher-order positive psychological construct integrating resilience, hope and optimism can be established. More importantly, the three state-like positive psychological variables are malleable and open to change. Resilience can be enhanced through interventions at different levels. Based on the theory of social determinants of health, Khanlou and Wray proposed the concept of a whole community approach, which indicates that resilience interventions should not be limited to family, school settings, community and social factors are more important in resilience building [52]. Luthar and Brown have pointed out that relationships are the “roots” of resilience: the presence of support, love, and security fosters resilience by reinforcing people's innate strengths [53]. Interventions for successfully raising hope in both clinical and non-clinical settings have been developed [54, 55]. Identifying potential pathways to realizing goals, positively interpreting failures, psycho education are among the commonly used strategies to increase positive emotions and expectancy, which enables one to actively engage in goal pursuits. These interventions have resulted in higher confidence in one’s ability to achieve goals and increased capacity for identifying environmental resources for goal accomplishment [56]. Although dispositional optimism is relatively stable, research indicates that optimism is positively correlated with coping strategies [57, 58], which can be cultivated through psychosocial intervention such as cognitive behavior therapy [59, 60]. Additionally, in a 10-year longitudinal study of law students, Suzanne confirmed that social network growth did predict increases in optimism [61]. Therefore, integrated interventions to increase resilience, hope, and optimism can be conducted to prevent and treat depression in medical students.

Several limitations of the present study have to be acknowledged. First, due to the nature of cross-sectional study, we were unable to draw causal relations among the study variables. The findings from the study should be confirmed by prospective cohort studies in the future. Second, all data were obtained through self-reported questionnaires, which could introduce response bias. The participants might have underestimated or overestimated the relationship between depression and psychological variables. Thirdly, although some socio-demographic factors were included in the study, other factors, such as family history of depression, were not considered. Fourthly, given the study sample, the generalization of the results should be taken with caution. More research should be conducted in other cultures.

## Conclusion

This study showed high prevalence of depressive symptoms among Chinese medical students (66.8 %). Older students, male students and students in 5-year programs had a higher prevalence of depressive symptoms relative to their counterparts. The three positive psychological variables of resilience, hope and optimism as a whole had significant associations with depressive symptoms in the students. Medical colleges and universities in China should give their immediate attention to the prevention and treatment of depressive symptoms among the students. Intervention strategies that focus on enhancing the positive psychological constructs of resilience, hope and optimism can be integrated into the depression prevention and treatment programs.

## Abbreviations

CES-D: Center for Epidemiologic Studies Depression Scale
RS-14: Wagnild and Young Resilience Scale-14
ADHS: Adult Dispositional Hope Scale
LOT-R: Life Orientation Test-Revised
WMA: World Medical Association
SPSS: Statistical Package for the Social Sciences
SD: Standard deviation
ANOVA: Analysis of variance
